# Vegetative pyoderma gangrenosum: A prospective case series evaluating clinical and patient-reported outcomes

**DOI:** 10.1016/j.jdcr.2026.04.019

**Published:** 2026-04-20

**Authors:** Katelyn Downey, Richard Zhang, Shannon K. Throckmorton, Jordan Gillespie, Jaclyn Roland-McGowan, Jesse J. Keller, Alex G. Ortega-Loayza

**Affiliations:** a Department of Dermatology, Oregon Health & Science University, Portland, Oregon; b Larner College of Medicine, University of Vermont, Burlington, Vermont

**Keywords:** patient-reported outcomes, vegetative pyoderma gangrenosum, wound healing

Vegetative pyoderma gangrenosum (vPG) is a rare and poorly understood variant of pyoderma gangrenosum (PG), first described in 1988.^1^ Compared with classic PG, vPG has traditionally been described as less aggressive, with less pain, fewer systemic associations, and a lower likelihood of requiring systemic treatment. Lesions usually present on the trunk and are characterized by superficial, verrucous plaques associated with granulomatous histologic findings that can develop sinus tracts leading to cribriform scarring.^2^ Because these features overlap with infectious and other inflammatory dermatoses, the diagnosis and management of vPG are challenging.

To better characterize vPG, we conducted a prospective study at Oregon Health & Science University between December 2019 and May 2025. Standardized clinical data were collected at patient encounters, and as part of routine care, patients completed questionnaires at check-in. These included the Skindex-Mini (0-100 scale; symptom, emotion, and function domains) and Numerical Rating Scales (NRS; 0-10) for pain and pruritus.^3^^,^^4^ Disease severity was assessed by patients using the Patient Global Assessment and by investigators through the Investigator Global Assessment.

Of 266 subjects evaluated for suspected PG, 9 were initially identified as possible vPG during routine clinical care by a dermatologist with PG expertise (A.G.O.L.). Evaluation incorporated a standardized PG workup, including prospective calculation of PARACELSUS score,^5^ assessment of clinical morphology and disease behavior, exclusion of infectious mimickers, and histopathologic evaluation when available. These 9 cases subsequently underwent independent, blinded review by a second dermatologist with PG expertise (J.K.), resulting in consensus classification of 7 patients (2.6%) as possible vegetative PG (Table I). Given known limitations of existing diagnostic criteria in atypical PG variants, no single diagnostic criterion was required for inclusion, and the term “possible vegetative PG” was used to reflect this diagnostic uncertainty and the absence of validated diagnostic criteria specific to vPG.

**Table I:** Clinical and diagnostic characteristics of patients with vegetative pyoderma gangrenosum

Case	Age (y); Sex	PG-associated comorbidities	Conditions affecting wound healing	Lesion location/number	Target lesion size (cm^2^)	Biopsy (Y/N)	Histopathology	Pathergy history (Y/N)	PARACELSUS score (baseline)
1	35; F	UC	None	Legs/Multiple	120.0	N	NA	N	2∗
2	71; F	MGUS, HS	None	Legs/Multiple	93.5	Y	Granulomatous dermatitis with pseudoepitheliomatous hyperplasia	Y	20
3	62; M	None	Venous insufficiency, T2DM, HTN	Legs/One	11.9	Y	Mixed inflammatory dermatitis with inflamed follicular sinus	N	11
4	40; M	PAPASH syndrome, PsA	HTN	Legs/Multiple	67.5	Y	Suppurative follicular inflammation	N	9
5	59; M	None	T2DM, HTN	Legs/Multiple	195.0	Y	Suppurative granulomatous dermatitis with neutrophils	Y	19
6	88; M	None	T2DM, HTN	Trunk/One	37.5	Y	Suppurative follicular inflammation	Y	11
7	37; F	HS	Obesity, venous insufficiency	Legs/Multiple	4.9	N	NA	N	8∗

*HS*, Hidradenitis suppurativa; *HTN*, hypertension; *MGUS*, monoclonal gammopathy of undetermined significance; *NA*, not applicable (biopsy not performed); *PAPASH*, pyogenic arthritis, pyoderma gangrenosum, acne, hidradenitis suppurativa; *PsA*, psoriatic arthritis; *T2DM*, type 2 diabetes mellitus; *UC*, ulcerative colitis.

∗ Patient was receiving systemic immunosuppressive therapy at time of baseline assessment.

Skin biopsy findings supported the diagnosis in 5 cases; 2 patients did not undergo biopsy. In one patient with PAPASH syndrome, histopathologic findings included suppurative follicular inflammation, which may reflect overlap with underlying hidradenitis suppurativa rather than features specific to vPG (Table I). ANCA serologies were obtained in 3 of 7 patients. One patient had positive proteinase 3 antibodies and systemic inflammatory manifestations; however, rheumatologic evaluation and extensive tissue sampling did not demonstrate evidence of ANCA-associated vasculitis, and pulmonary findings were attributed to ulcerative colitis–associated disease. Urine toxicology screening was obtained in one patient and was negative for cocaine use.

Lesions were located on the lower extremities in 6 patients and on the trunk in one (Fig 1). Patients had a mean age at diagnosis of 60 y (SD 19), a median time to diagnosis of 12 m (range 0.5-42 months), and a median lesion size of 67.5 cm^2^ (range 4.9-195 cm^2^). Two patients had a single lesion, while 5 had multiple (Table I). In patients with multiple lesions, all present at the time of diagnosis demonstrated similar morphology and clinical behavior and were considered consistent with vPG. For longitudinal assessment, the target lesion was defined as the largest lesion present at the time of diagnosis.

**Fig 1:**
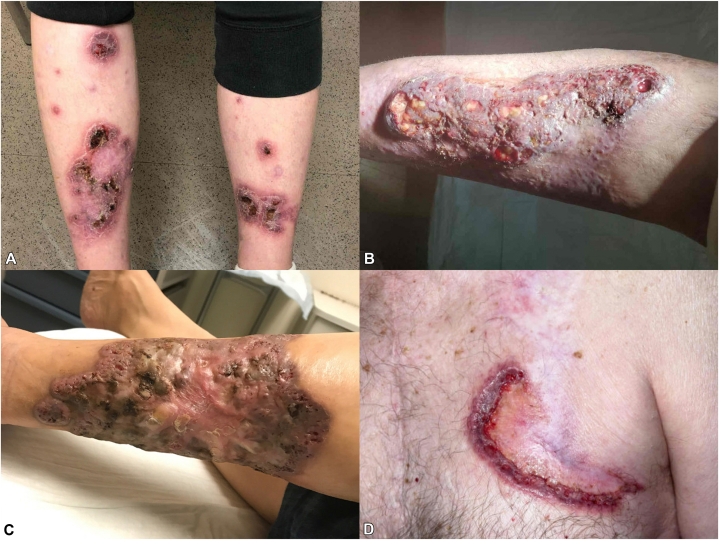
Representative baseline presentations of vegetative pyoderma gangrenosum. **A,** Case 1, Multifocal lower extremity involvement with superficial vegetative plaques. **B,** Case 2, large vegetative plaque with ulceration on the lower extremity. **C,** Case 5, extensive vegetative plaque on the lower extremity with exophytic granulation tissue. **D,** Case 6, truncal lesion demonstrating superficial ulceration with erythematous borders.

Baseline pain NRS was modest (median 4; range 1.5-9), whereas baseline pruritus was frequently reported, with a median NRS of 5 (range 0-7). Among patients with baseline Skindex-Mini data, medians indicated a high disease burden: 100 for symptoms, 67 for emotions, and 100 for function. Upon healing, defined as complete re-epithelialization, patients reported improvements in NRS scores (median pain 0.25, range 0-3; median pruritus 3, range 0-7). Last-follow-up Skindex-Mini was available for only one healed patient, who reported 0 across all domains.

Treatment responses were variable. Patients received corticosteroids (*n* = 6), biologics (*n* = 6), topical calcineurin inhibitors (*n* = 4), topical corticosteroids (*n* = 4), intralesional corticosteroid injections (*n* = 3), doxycycline (*n* = 2), dapsone (*n* = 2), colchicine (*n* = 2), JAK inhibitors (*n* = 1), methotrexate (*n* = 1), and cyclosporine (*n* = 1) with variable responses.

Four patients healed with a median time to target lesion resolution of 5.1 months (range 1.5-10 months). Among patients whose target lesion achieved complete re-epithelialization, one remains in remission on maintenance infliximab. In another patient, the target lesion healed; however, additional vegetative plaques present at the time of diagnosis did not fully resolve, and the patient continues on infliximab with improvement. Two patients experienced recurrence of classic PG following healing of the target lesion. In one patient, recurrence occurred 6 m after healing and subsequently resolved after 4.5 months with colchicine and topical corticosteroids. In the other, recurrence developed 0.75 months after healing and resolved after 2.5 months with infliximab. One patient died prior to healing 16.5 months after diagnosis due to acute respiratory failure unrelated to vPG. Additionally, one patient developed chronic vPG requiring ongoing biologic maintenance therapy, and one was lost to follow-up.

The patients in this study did not fully align with traditional descriptions of vPG. Although lesions were less painful than classic PG, most were multifocal and located on the lower extremities. All patients had at least one systemic comorbidity associated with PG or impairing wound healing. While vPG has been described as responsive to conservative therapies, including topical or intralesional steroids and antimicrobial agents, all patients in this cohort required systemic immunosuppression. As a referral center, we likely see more treatment-refractory cases; however, the need for systemic therapy and presence of recurrences suggest that vPG may not be as treatment-responsive as previously thought.

Patient and investigator assessments of disease severity were not uniformly aligned, with several cases demonstrating discordance between PGA and IGA ratings (Table II). This variability highlights the importance of incorporating patient-reported outcomes when evaluating the full disease burden of vPG.^6^

**Table II:** Disease severity, patient-reported outcomes, and clinical course in patients with vegetative pyoderma gangrenosum

Case	PGA (baseline, LFU)	IGA (baseline, LFU)	Pain (baseline, LFU)	Pruritus (baseline, LFU)	SDM-S (baseline, LFU)	SDM-E (baseline, LFU)	SDM-F (baseline, LFU)	Wound age at diagnosis (mo)	Target lesion outcome at LFU	Time to healing (mo)	Recurrence (Y/N)
1	—, —	Almost clear, clear	(1.5, 0.5)	—	—	—	—	20	Healed	4.5	N
2	Severe, severe	Severe, moderate	(9, 0)	(5, 3)	(100, 33)	(67, 0)	(100, 67)	42	Healed	10	N
3	Severe, clear	Severe, clear	(3, 0)	(5, 0)	(50, 0)	(17, 0)	(100, 0)	10	Healed	5.75	Y (classic PG)
4	Severe, severe	Moderate, severe	(5, 9)	(5, —)	(100, 100)	(83, 100)	(50, 100)	12	Unhealed	NA	NA
5	—, Almost clear	Severe, almost clear	(9, 0)	(—, 8)	(—, 0)	(—, 0)	(—, 0)	8	Lost to follow-up	NA	NA
6	Severe, moderate	Moderate, severe	(4, 4)	(0, 0)	(67, 50)	(67, 17)	(67, 33)	42	Died prior to re-assessment	NA	NA
7	Moderate, —	Moderate, clear	(3, 3)	(7, 7)	(100, —)	(100, —)	(100, —)	0.5	Healed	1.5	Y (classic PG)

LFU represents the date of target lesion healing or, for unhealed cases, the last available clinical follow-up for the target lesion. For patients with recurrent disease, LFU and outcomes refer to the target lesion and do not reflect subsequent lesions.

*IGA*, Investigator Global Assessment; *LFU*, last follow-up; *NA*, not applicable (target lesion not healed); *PGA*, Patient Global Assessment; *SDM-E*, Skindex-Mini Emotion; *SDM-F*, Skindex-Mini Function; *SDM-S*, Skindex-Mini Symptom; —, data unavailable.

Pruritus has not been extensively characterized in vPG; however, itch has been reported in patients with PG within broader cohorts of chronic wounds. In a prospective study of pruritus in chronic leg ulcers that included 3 patients with PG, itch was observed in 2 cases.^7^ Although limited by small sample size, the frequent reporting of pruritus in our vPG cohort is consistent with these prior observations and suggests that itch may represent an under-recognized component of disease burden that warrants further study. In this context, prominent pruritus relative to pain may be a clinically relevant feature in some cases of vPG**,** though larger studies are needed to determine whether this observation has diagnostic significance.

Treatment approaches in this cohort further reflect the complexity of disease management. Several systemic therapies, including corticosteroids, biologics, methotrexate, cyclosporine, and JAK inhibitors, were selected for their activity against both vPG and coexisting inflammatory comorbidities, reflecting real-world management in a tertiary referral setting.

This study has several limitations. ANCA serologies and urine toxicology were not uniformly obtained in all patients, reflecting real-world clinical practice and a limitation of this cohort. Facial superficial granulomatous PG was not represented in this cohort, as no patients with facial wounds meeting criteria for vPG were identified during the study period. As such, these findings may not be generalizable to facial superficial granulomatous PG, which has been described as a distinct clinical entity.

Although these conclusions are limited by the small sample size of 7 patients, the data challenge the perception of this subtype as indolent and add valuable information from the patient perspective that has not been described in the 91 previously published vPG cases.^2^ Future studies are needed to develop specific diagnostic criteria and better characterize the clinical course of this rare PG subtype.

## Conflicts of interest

Dr Ortega-Loayza serves as an associate editor for *Dermatology (Karger)* and editorial board member of the *American Journal of Clinical Dermatology*. Additionally, he is a consultant for Genentech and Guidepoint and an advisor to Bristol Meyer Squibb, Boehringer Ingelheim, and Janssen. Dr Ortega-Loayza has received research grants from Eli Lilly, Janssen, Incyte, and Pfizer. He is supported by NIH NIAMS R01 AR083110. Dr Keller is a speaker for Galderma. Authors Downey, Zhang, Gillespie, McGowan, and Dr Throckmorton have no conflicts of interest to declare.
